# The Story of the Insane from Year to Year

**Published:** 1902-02-15

**Authors:** 


### THE STORY OF THE INSANE FROM YEAR TO YEAR.

FOURTEENTH ANNUAL SERIES.

### EAST SUSSEX ASYLUM AT HAYWARD'S HEATH.

ON the last day of 1900 this asylum contained 967 patients, having increased its numbers by 12 during the year. There were 228 first admissions, 37 readmissions, 91 recoveries, 38 discharged relieved, and 91 deaths. The average daily number resident was 970, and 1,218 persons were under treatment during the year. The recoveries stand at 35·8 per cent. of the admissions, and the deaths at 9 3 per cent. on the average number resident. Cerebral and spinal diseases account for 51 of the deaths, consumption for 2, other lung diseases for 14, and colitis for 1. Moral causes are given as having either predisposed to, or excited, the insanity in 27 of the year's admissions; intemperance in drink in 28, hereditary influence in 66, congenital defect in 13, and in 71 no cause was known. For a long time there has been a necessity for further accommodation in this asylum, and temporary buildings for 100 patients have been erected and filled; but still more room was wanted, and accommodation was found for 30 female patients in the Staffordshire Asylum. The visitors are fortunate in having to pay only 15s. a week each for those boarded out. Scarlatina broke out and attacked 1 medical officer, 7 attendants, and 8 patients. Dr. Saunders, the medical superintendent, resigned during the year, his extended leave of absence having failed to re-establish his health. While regretting the cause of resignation we congratulate Dr. Saunders on having received a fair pension for his 12 years' services. Superannuation allowances were also granted to James Barker, Thomas Puddington, and James Pilkington. Dr. Walker was promoted to the superintendency, Mr. Planck was made senior assistant, Mr. Lewis Cook second assistant, and Mr. H. J. Foster third assistant. The asylum is now very strongly manned from a medical point of view, and much scientific work may be confidently looked for. The visitors express their appreciation of the work of the new medical superintendent. The weekly rate of maintenance was 9s. 11⅝d. per head.

### WONFORD HOUSE HOSPITAL FOR THE INSANE, EXETER.

HERE the numbers rose during the year from 127 to 131. There were 26 first admissions, 8 readmissions, 11 recoveries, 7 discharged relieved, 8 not improved, and 4 died. The average number resident was 131, and the total number of persons under care was 158. The recoveries stand at the creditable percentage of 52·3 of the admissions, and the deaths are very low, being only 3 per cent. of the average number resident. Intemperance in drink accounted for 6 of the year's admissions, and hereditary influence for 7. In

5 no cause was known. We have much pleasure in noting that there has been no appreciable falling off in the amount of charitable work done by this hospital. Unlike some of its congeners its main object is not to secure a large number of patients who pay very high rates, and who would be as well or better in private asylums, but to help those who are unable to command such accommodation. Three are maintained without payment, and 58 at reduced payments, of whom 10 pay less than a pound a week. Dr. Deas thinks that insanity, apart from certain forms of brain disease, does not tend to shorten life, and he instances in proof of this that of the total of 131 patients 56 were over 60 years of age, 16 of these being between 70 and 80, and 7 were upwards of 80. In truth the chronic lunatic of the present day, if not given to fretting, has a very pleasant, equable sort of life, and is in every way well cared for, hence his chances of old age are considerable. The chaplain, Mr. Kitchin, after 24 years' service, retired on a pension of £100 a year, and the committee very wisely appointed the vicar of the parish as chaplain. By this means the work will probably be better done at a lower sum than if a private chaplain were appointed. Indeed it is an example which might advantageously be followed in many of our county asylums. The average weekly cost of maintenance was £1 14s. 10d.

